# Plants and water in a changing world: a physiological and ecological perspective

**DOI:** 10.1007/s12210-022-01084-7

**Published:** 2022-08-13

**Authors:** Roberto Caferri, Roberto Bassi

**Affiliations:** Dipartimento di Biotecnologie, Università di Verona, Verona, Italy

**Keywords:** Water use efficiency, Carbon dioxide, Changing environment, Plant productivity, Transgenic crops

## Abstract

The reduction of greenhouse gases (GHGs) emission by replacing fossil energy stocks with carbon–neutral fuels is a major topic of the political and scientific debate on environmental sustainability. Such shift in energy sources is expected to curtail the accumulation rate of atmospheric CO_2_, which is a strong infrared absorber and thus contributes to the global warming effect. Although such change would produce desirable outputs, the consequences of a drastic decrease in atmospheric CO_2_ (the substrate of photosynthesis) should be carefully considered in the light of its potential impact on ecosystems stability and agricultural productivity. Indeed, plants regulate CO_2_ uptake and water loss through the same anatomical structure: the leaf stomata. A reduced CO_2_ availability is thus expected to enhance transpiration rate in plants decreasing their water use efficiency and imposing an increased water demand for both agricultural and wild ecosystems. We suggest that this largely underestimated issue should be duly considered when implementing policies that aim at the mitigation of global environmental changes and, at the same time, promote sustainable agricultural practices, include the preservation of biodiversity. Also, we underlie the important role(s) that modern biotechnology could play to tackle these global challenges by introducing new traits aimed at creating crop varieties with enhanced CO_2_ capture and water- and light-use efficiency.

## Introduction

Life on earth is carbon-based. Of the estimated 550 gigatons of carbon in the biosphere, ≈ 82% is stored in plant biomass (Bar-On et al. 2018). It follows that photosynthetic organisms are the main contributors to the ecological stability of the planet.

**Fig. 1:**
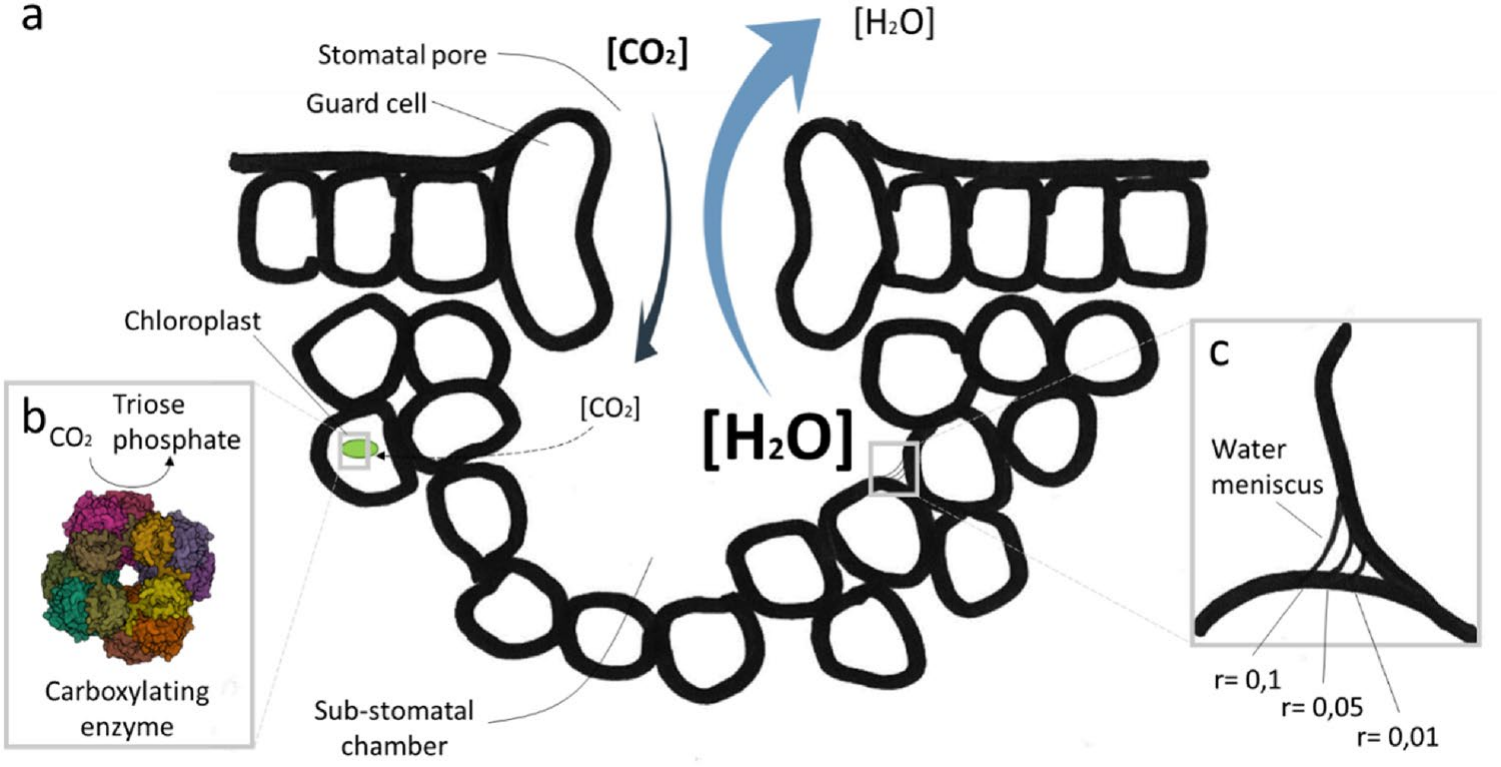
Representation of gas dynamics across the stomatal pore and CO_2_ diffusion inside plant cells. **a** CO_2_ and H_2_O flow through the stomatal pore following their gradient concentration. **b** The carboxylating enzyme RuBisCO (PDB file: 1RSC) located in the chloroplast is shown. RuBisCO activity sustains substrate consumption, thus lowering CO_2_ concentration in the sub-stomatal chamber. This enzymatic activity maintains the gas concentration gradient which, in turn, drives CO_2_ absorption. **c** The water meniscus radius changes according to the leaf water potential. In the presence of high evapotranspiration rates, the water meniscus radius decreases, thus imposing a negative tension force that drives water transport rate from roots to leaves through xylem vessels

Plant life relies on few but extremely crucial environmental parameters: CO_2_ and water availability, sunlight, temperature, and minerals in soils. Water availability is the most limiting factor for plant development and biomass productivity. However, plants are faced with a physiological dilemma. The uptake of CO_2,_ the primary substrate of photosynthesis, and water transpiration occur via the same anatomical structure: the stomatal pore. Stomata consist of pairs of guard cells that enable the opposite movement of CO_2_ and water within sub-stomatal chamber (Fig. 1a). The opening and closure of stomata is subjected to a finely tuned regulation, which is required to optimize gaseous influx while minimizing water loss between the atmosphere and the leaf tissue.

During evolution, land plants have developed survival strategies to enhance their CO_2_ fixation and water-use efficiency (WUE), *i.e.* the amount of water evaporated per weight of biomass accumulated during growth. Interestingly, one of the main evolutionary driving forces behind these physiological adaptations was a gradual decrease in the atmospheric CO_2_ concentration (Christin et al. 2008).

Anthropogenic carbon emissions significantly influence the atmospheric gas composition and thus are expected to affect plant fitness on relatively short time scales. For instance, the industrialization process marked the start of a steady increase in the atmospheric CO_2_ concentration, with an estimated value of 278 ppm at the onset of the industrial revolution and of 408 ppm being registered recently (https://ourworldindata.org/atmospheric-concentrations). As it will be discussed later, this rise in atmospheric CO_2_ levels could potentially relax the selective pressure for evolving additional CO_2_ capture mechanisms.

However, the rise in CO_2_ levels constitute a matter of concern among the scientific community. CO_2_ absorbs infrared radiation and thus contributes to the warming effect of the atmosphere (Arrhenius 1896; Wei et al. 2018). Accordingly, a shared consensus view supports the replacement of conventional fossil fuels with so-called carbon–neutral energy sources to slow down this process.

Despite its infamous role in global warming, CO_2_ is the substrate of photosynthesis which plants utilize to build their organic constituents. As shown by free-air CO_2_ Enrichment (FACE) experiments, local increases in ambient CO_2_ concentration positively correlate with plant biomass accumulation in several species via a concomitant enhancement of photosynthetic light- and water-use efficiency (Ainsworth and Long 2005; Leakey et al. 2009) with reported enhancements of plant biomass yield up to 42% in *Gossypium* (Mauney et al. 1994).

On the contrary, it could be speculated that marked reductions of CO_2_ might promote desertification because of heightened plant water demand, finally threatening food security and ecosystem fitness on Earth.

A clear understanding of the physiological relationship between CO_2_ uptake and water use in plants is thus essential to predict the potential impact of environmental changes on vegetation fitness, but also to guide biotechnological approaches towards the development of crop varieties with enhanced stress tolerance.

Below, we report on the role of CO_2_ and water in plant life and how a change of CO_2_ levels could alter their delicate balance.

### Water is essential for plant life

Water is the universal solvent for the metabolism of all living organisms. Almost all biochemical reactions involving proteins, nucleic acids and metabolites occur in water-based solvents. In plants, however, water plays additional roles both at the subcellular and extracellular levels. As a defining trait, plant cells include a large water-filled compartment—the vacuole—which, besides being involved in metabolic functions, contributes to building cellular turgor pressure. At tissue scale, water is involved in nutrient absorption from the soil via the water-permeable root system and their transport through a hydraulic tubing (the xylem) towards leaves, where it is finally lost by transpiration.

From a biophysical perspective, water is the substrate of light-driven photosynthetic reactions, serving as the source of electrons that linearly flow through thylakoid complexes and sustain the synthesis of the high energy molecules NADPH and ATP used by the Calvin Cycle for carbon fixation (Bassham et al. 1950). A valuable by-product of the water-splitting event is molecular oxygen, which is released in the atmosphere and, in turn, enables cellular respiration of heterotrophic species.

**Fig. 2:**
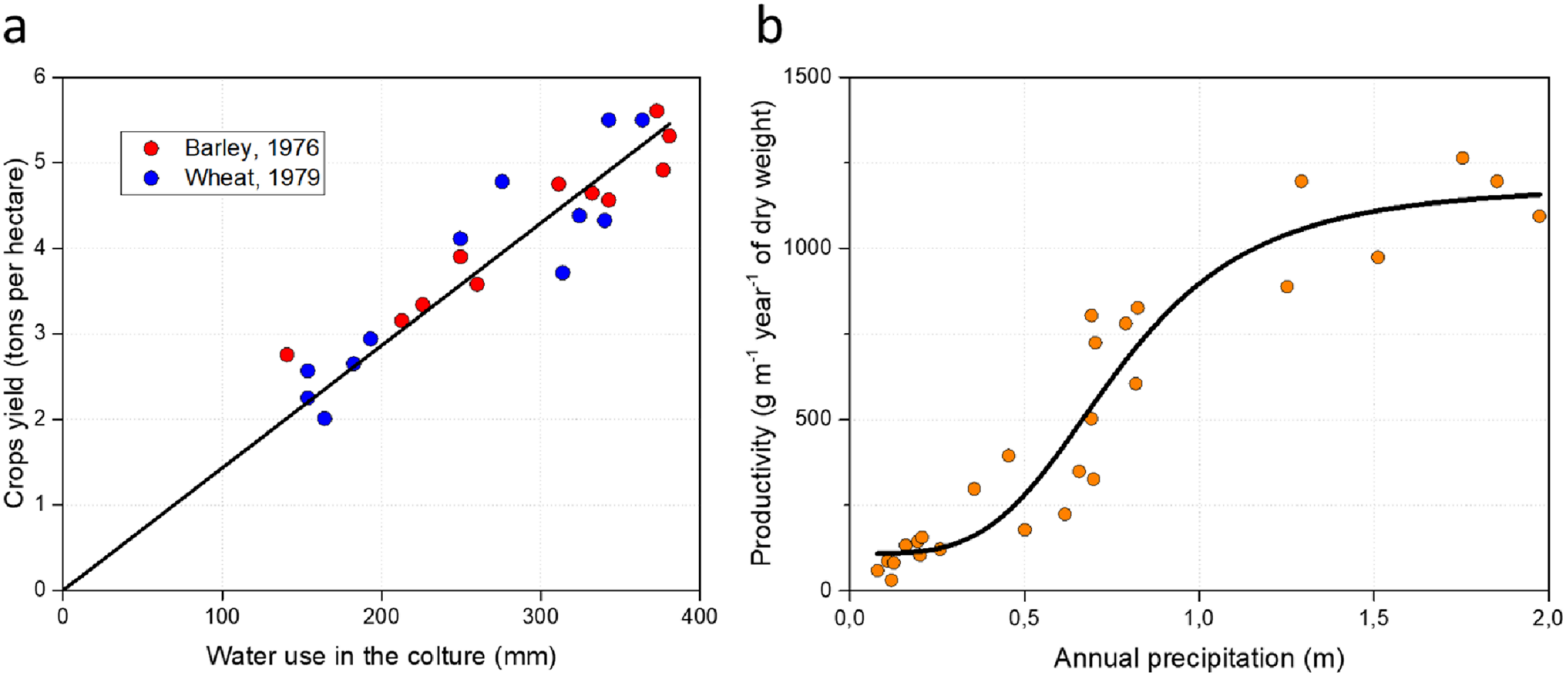
Correlation between water supply and plant productivity. **a** The graph shows the yields of barley (red points) and wheat (blue points) under different water regimens (expressed as millimiters of water used during the entire productive cycle; data extracted from Day et al. 1978; and Innes and Blackwell 1981). **b** Ecosystem productivity related to the annual precipitations. Total productivity positively correlates with water abundance for values below 1 m per year (data extracted from Whittaker 1970)

Water availability, which is primarily regulated by ambient temperature, is the main limiting factor for plant growth. Planetary climatic areas are defined by their water abundance. Deserts, where water is in shortage, support little vegetation (Gaston 2000). Cold environments, where water availability is also limited, display poor biodiversity and low density. Instead, temperate (and tropical) zones, where water is available throughout the year, are characterized by rich and diverse ecosystems. On a global scale, massive water amounts are required to sustain plant growth. It has been estimated that the fixation into biomass of 1 mol of CO_2_ requires, on average, the loss of 400 mol of H_2_O (Leakey et al. 2019). Only a few species, equipped with peculiar water storage systems, have lower water demand such as CAM plants (Luttge 1987). Water availability is crucial for agricultural yield and food security. In 1992, Jones described the relationship between irrigation regimes and barley grain yield using data collected since 1976. A strong correlation emerged between used water and yield, and the same observation was verified for wheat (Jones 1992; Day et al. 1978; Innes and Blackwell 1981) (Fig. 2a). At ecosystems level, plant biomass productivity also correlates with annual precipitation values. However, in this case, the trend is only conserved until 1 m/year; beyond this value the enhancement effect was saturated (Whittaker 1970) (Fig. 2b). Overall, these observations clearly indicate that primary productivity through carbon fixation is strongly dependent on water availability.

### Water and CO_2_ uptake in higher plants

Upon absorption from soil water can travel several routes to reach the inner core of the root vascular system: apoplastic, symplastic and transmembrane. After crossing the watertight Casparian strip surrounding the radical stele, water enters the xylem which consists of specialized elongated cell types: tracheids and vessel elements. These structures are typical of higher plants and have both primary and secondary walls. Xylem vessels can transport water with high efficiency through plant tissues and the water movement is explained by the “cohesion-tension” theory (Tyree 1997). Xylem vessels consist of dead cells which expose their hydrophilic cellulosic surface establishing cooperative hydrogen bonds with water molecules that flow upwards following the negative pressure generated in the mesophyll. In particular, the negative pressure originates on the leaves at the interface between the air-exposed surface of mesophyll cells within the substomatal chamber. This generates a water flux from the intercellular space towards the cellulosic cell wall. Following transpiration, the curvature radius of the meniscus of the residual water accumulating in the leaf interstices decreases, producing a negative pressure (tension) (Fig. 1c). The relationship between meniscus radius curvature and hydrostatic pressure ($\Psi p$) is given by the formula:

$$\Psi p= -\frac{2T}{r}$$

where, *T* is the water surface tension (estimated as 7.28 × 10^–8^ MPa m) and *r* is the curvature radius of water meniscus. Thus, water flows from roots to leaves without energy consumption. Stomatal conductance is regulated via signaling networks that rely, among other factors, on the hormone abscisic acid (ABA) (Cutler et al. 2010). ABA is also involved in the response to freezing and salinity stress and upon drought stress its intracellular level increases to prevent critical water loss (Tuteja 2007).

Since CO_2_ uptake also occurs via stomatal pores, their opening is unavoidably accompanied by a net water loss via a process known as evapotranspiration. Fick’s laws of diffusion (1855), helps to explain the dynamics of gas flow through stomata. Fick’s first law describes the relation between the diffusive flux and the concentration gradient of a substance, which is given by the following formula:

$$J= -D \frac{\mathrm{d}\varphi }{\mathrm{d}x}$$

According to the relation, the amount of CO_2_ or H_2_O that flows through the stomata, which is represented by the diffusion flux (*J*), can be estimated by using the diffusion coefficient (*D*), the gas concentration in- and outside the leaf (d*φ*) and the distance (d*x*). Broadly, fluxes move from highly concentrated regions to low concentrated ones. At the level of the substomatal chamber water and CO_2_ display opposite gradients. The CO_2_ gradient depends on the activity of the carbon fixing reactions which consumes it within cells (Fig. 1b). However, the external CO_2_ concentration is very low (≈ 0.04%) and thus only a relatively small gradient exists for this gas. Water, instead, is at saturation point in the stomatic chamber and thus is subjected to a steeper gradient, which is further maximized in dry air conditions (Monteith and Unsworth 2013). In conclusion, almost 95% of the water extracted from the soils through the root system evaporates while, conversely, a much smaller amount of CO_2_ is incorporated.

As sunlight fuels photosynthesis, plants open their stomata. CO_2_ enters the substomatal chamber and dissolves in the water soaking cell walls, finally reaching the chloroplast stroma. Here, multienzyme complexes are responsible for its incorporation into triose phosphate sugars via the Calvin–Benson–Bassham (CBB) cycle. The CBB cycle entails three enzyme-catalyzed steps: carboxylation, reduction, and substrate regeneration. The rate-limiting steps of this process is implemented by the enzyme Ribulose-1,5-Bisphosphate Carboxylase/Oxygenase (RuBisCO), which catalyzes the first committed step of the Calvin cycle responsible for the incorporation of CO_2_ in the acceptor ribulose 1,5-bisphoshate (Cleland et al. 1998).

Plants CO_2_ assimilation mechanism display an additional inherent physiological flaw: RuBisCO, despite being responsible for the fixation of over 90% of the inorganic carbon globally (Erb and Zarzycki 2018), cannot efficiently discriminate between its primary substrate CO_2_, and oxygen, which is highly enriched within the chloroplast as the main by-product of Photosystem II activity (Steiger et al. 1977; Miziorko and Lorimer 1983). This property is particularly disadvantageous and creates competitive inhibition of RuBisCO. While the overall reaction between RuBisCO and CO_2_ produces two 3-phosphoglycerate (3PG) molecules that can be used for sugar synthesis, reaction with O_2_ leads to one 3PG and one glycolate molecule. This latter is a C2 oxygenated compound which cannot be used in sugar synthesis and is also toxic because of the strong acidification of chloroplast stroma it induces (Wada et al. 2020). The energy loss caused by glycolate metabolism is only partially attenuated by a complex metabolic pathway known as photorespiration (Wingler et al. 2000) which recovers ≈ 75% of absorbed carbon. However, photorespiration dramatically decreases carbon fixation efficiency and, consequently water-use, especially at low atmospheric CO_2_ levels. On average, it is estimated that every four catalytic cycles by RuBisCO, one is wasteful (Leegood 2007). It can be asked why plants fix CO_2_ via RuBisCO instead of using a different carboxylating enzyme with higher substrate specificity. This is rationalized by the fact that oxygen accumulated in the atmosphere as product of photosynthetic processes, and therefore it could not act as a negative selective pressure during the evolution of carbon fixation. This was only possible when RuBisCO-based metabolism was already established as the only pathway in low CO_2_ conditions. Indeed, there is phylogenetic evidence that RuBisCO was placed under selective pressure at least during two great oxygenation events (Savir et al. 2010; Erb and Zarzycki 2018), which resulted in the evolution of isoforms with higher ability to discriminate between CO_2_ and O_2_ (Whitney et al. 2011). Despite reaching the 1:100 (O_2_:CO_2_) specificity being a good performance, it is not yet optimal, especially if one considers that the current concentration of oxygen in the air is ≈ 21%, while CO_2_ only reaches ≈ 0.04%. Thus, the O_2_/CO_2_ ratio is approximatively 500 in the best conditions. Within a crop field CO_2_ is even more depleted from canopy air due to photosynthetic activity (Seibt et al. 2004).

### Plant physiological adaptations to maximize CO_2_– and water-use efficiency

As emerged from geological records, CO_2_ levels fluctuated significantly during the last 35 million years showing a decreasing trend (Pagani et al. 2005). This situation impinged on photosynthetic organisms to develop physiological adaptations to optimize water use while maintaining efficient CO_2_ fixation. One remarkable example of evolutionary adaptation is the C4 photosynthetic metabolism. C4 plants exploit a CO_2_-concentrating mechanism which enriches this substrate around the active sites of RuBisCO.

The C4 pathways relies on a peculiar leaf anatomy (the Kranz anatomy) that enables the spatial partitioning of enzyme activity in two tissue types: the bundle sheath and mesophyll cells. Operationally speaking, gaseous CO_2_ entering the stomata of C4 plants dissolves within the cytoplasm of mesophyll cells, where is incorporated by the enzyme Phosphoenolpyruvate carboxylase (PEPC) into the C4 organic compound oxalacetate. It is worth noting that the substrate for the PEPC is HCO_3_^−^ rather than CO_2_, thus avoiding the competition with O_2_, which is instead the case for RuBisCO during its oxygenating activity leading to photorespiration (Spreitzer and Salvucci 2002). Oxalacetate is then transported in the chloroplasts of bundle-sheath cells from which CO_2_ is released (Schlüter and Weber 2020). This functional compartmentalization enhances the carboxylating efficiency of RuBisCO by creating a CO_2_-rich/O_2_-deplete environment, reducing the wasteful photorespiratory cycle. At the same time, by increasing the local CO_2_ levels, this mechanism contributes to the water-conserving phenotype of C4 species.

**Fig. 3:**
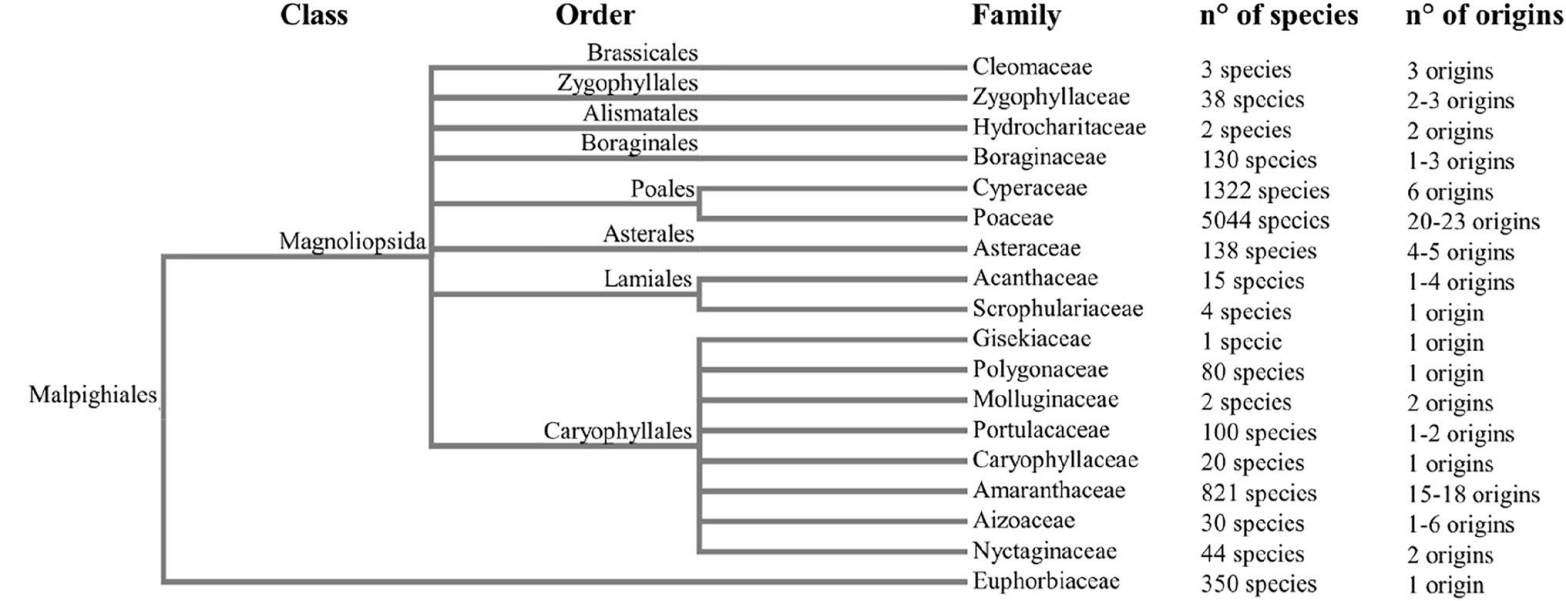
Phylogenetic tree displaying plant families in which species with C4 carbon assimilating metabolism has been identified. Number of C4 species for each taxonomic rank and number of independent origin events for appearance of C4 traits for each family are indicated.

It has been estimated that this variant of CO_2_ fixing metabolism has evolved at least 60 times independently over the last 35 million years (Sage 2004) (Fig. 3), thus representing a true example of convergent evolution.

It was suggested that this recurrent evolutionary adaptation was promoted by the existence of key genes responsible for the peculiar C4 trait already in a common ancestor within the plant lineage (Bräutigam et al. 2011). However, the selective pressure which established functional C4 phenotypes acted differentially on various ecological niches at different timepoints for each lineage by promoting a species-specific regulation of the expression of key genes (Hibberd and Covshoff 2010).

An even more extreme physiological adaptation to counteract water loss is the crassulacean acid metabolism (CAM), typical of species thriving in arid environments. CAM plants maximize their WUE via temporal segregation of CO_2_ capture and fixation (Osmond 1978). CAM plants keep their stomata open during the night and transiently store CO_2_ inside the vacuole as malate. During the day, CO_2_ is released from malate via decarboxylation and fixed by the CBB cycle. By keeping their stomata closed during the day, CAM plants avoid excessive transpiration promoted by high temperature (Hultine et al. 2019). This efficient water-saving phenotype comes at the cost of a slow growth rate due to a large energy investment required to sustain the CO_2_ concentrating mechanism.

### Will a changing atmosphere reshape ecosystems?

It is indisputable that the rising of temperatures favoured by increased CO_2_ levels imposes a higher water-demand for plants. This is because at higher temperatures CO_2_ is less soluble compared with O_2_, making lower the availability for carboxylation substrate and favouring photorespiration. Therefore, high stomatal conductance, which is promoted for CO_2_ uptake also increases water transpiration rates. Since C3 plants are extremely susceptible to warm and water scarce climates, this scenario will likely result in widespread desertification phenomena. On the contrary, this situation would have little impact on the fitness of C4 (and CAM) plant species, which already possess physiological adaptations to withstand temperature stress. Accordingly, it can be speculated that the distribution of C3 species will be significantly affected on a global scale, causing a reshaping of natural ecosystems in terms of species composition, including extinction events (Holsinger et al. 2019).

However, it should be considered that a CO_2_-enriched atmosphere can relieve the biochemical impediments of RuBisCO that restrain photosynthetic yield and primary metabolism. Furthermore, an increased CO_2_ availability promotes water conservation by reducing leaf transpiration and thus mitigates the impact of higher temperatures on vulnerable C3 species (Farquhar et al. 1980).

In support of this view, a recent ecosystem-scale FACE experiment conducted in a mature forest showed that the artificial CO_2_ enrichment resulted in a ≈ 12% higher carbon uptake used for primary biomass production (Jiang et al. 2020). However, this study highlighted that a significant portion of the supplied extra carbon was eventually emitted back via respiratory processes, thus questioning the real magnitude of the beneficial impact of CO_2_ elevation at ecosystem level.

Another recent analysis of remote sensing recordings spanning 38 years conducted over Eastern Australia reported a “greening trend” that correlates with the gradual rise in atmospheric CO_2_ levels, indicating a true carbon dioxide fertilization effect on ecosystem fitness (Rifai et al. 2022).

### Biotechnological approaches to tackle crop productivity in a changing environment

In the 1950s and 60 s the so-called “Green Revolution” unfolded under the guidance of American agronomist Norman Borlaug (Khush 2001), himself a disciple of Italian pioneer plant breeder Nazareno Strampelli (Scarascia Mugnozza 2005). This scientific-technological process relied on selective breeding approaches to introduce desirable traits in several crop species and was instrumental in achieving food security in many world areas chronically affected by hunger. The impact of this biotechnological shift is still felt today in modern agriculture as most high-yielding crop varieties developed at that time are still currently cultivated worldwide. A more recent biotechnological revolution started in the late 1980s, when plant molecular biologists successfully introduced heterologous genes in crop species, producing transgenic crops resistant to biotic threats and insensitive to herbicides (Prado et al. 2014). After almost three decades of extensive use, genetically modified crops have significantly enhanced agricultural output in many world areas (Raman 2017). However, ever since their introduction, GM crops are the constant subject of a public debates and, as consequence, their potential is restrained by strong political (and ideological) opposition despite the absence of experimental evidence supporting risks for the environment and human health. Indisputably, genetic engineering enabled the development of high yielding crops displaying superior stress tolerance, making GMOs irreplaceable tools to support food production for a growing world population. At the same time, the use of GM crops has a beneficial impact on biodiversity and on the stability of wild ecosystems by limiting the fraction of land occupied by agricultural practices, which is currently ≈ 38% (Krausmann et al. 2013) (https://ourworldindata.org/land-use). Today, scientists have a profound understanding of the genetic and biochemical basis that regulate plant physiology. Moreover, synthetic biology and genome editing techniques enable the introduction of new traits in plants and precisely modify their DNA sequences, including crop species (Lorenzo et al. 2022). It is not surprising that several attempts have been made already to enhance CO_2_- and water-use efficiency in plants through biotechnology.

The most ambitious project in this respect is the C4 Rice Consortium (Covshoff and Hibberd 2012; Kajala et al. 2011; von Caemmerer et al. 2012), in which scientists are trying to introduce the C4 photosynthetic metabolism in the staple C3 crop *Oryza sativa* to create new varieties with enhanced CO_2_-fixation and water-saving phenotypes.

This is a challenging approach since it requires modifications of the leaf anatomy and the compartmentalization of biochemical processes and could be obtained through an extensive rewiring of developmental regulatory gene expression networks. In parallel, more focused strategies are being pursued to improve the activity of enzymes involved in CO_2_ assimilation. A major target is the enhancement of the carboxylating turn-over rate of RuBisCO. A first achievement in this direction was accomplished by introducing in tobacco a faster cyanobacterial enzyme isoform with lower oxygenase activity which resulted in higher CO_2_ fixation rate compared with the native version (Lin et al. 2014). Additional routes have been proposed to solve these intrinsic functional photosynthetic bottlenecks. Viable options include the optimization of the expression of other CBB cycle enzymes and of regulatory elements of RuBisCO (Kubis and Bar-Even 2019). A particularly elegant synthetic biology approach aimed at reducing the negative impact of RuBisCO’s oxygenating activity on photosynthesis. This was achieved by engineering multiple alternative photorespiratory pathways in tobacco, two of which resulted in significantly higher biomass yield in field cultivation (South et al. 2019).

In addition to CO_2_- and water-use efficiency, plants could, in principle, be engineered to enhance their light use efficiency. It is well documented that plants are able to activate highly regulated molecular photoprotective mechanisms to safely dissipate energy under conditions of excessive irradiance (Bassi and Dall’Osto 2021). Although the conversion of light energy into heat via the so-called process of Non-Photochemical Quenching (NPQ) is needed to protect the photosynthetic apparatus from light-associated damage, this mechanism limits photosynthetic efficiency when it is activated in sub-stressful conditions. In a proof of principle approach Kromdjik and coworkers demonstrated that accelerating relaxation from the quenching (energy dissipating) state towards the unquenched (energy conserving) state, enhanced crop productivity in the field (Kromdijk et al. 2016).

Overall, these examples highlight the potential of rational genetic engineering to enhance plant fitness, especially in the context of predictable fluctuations of atmospheric CO_2_ levels.

## Outlook and perspectives

We conclude that the implementation of policies favoring a marked reduction of atmospheric CO_2_ concentration may counteractively impose additional physiological burdens on plant fitness by increasing their water demand. This situation would limit the use of water-scarce marginal lands for agriculture and exacerbate desertification effects, finally resulting in overall decrease of crop productivity (Tang et al. 2016).

Finally, it should be considered that a marked reduction of GHG emissions is likely to result in a higher transmission of solar irradiance with a consequent enhanced warming over lands. Such so-called brightening effect was already registered in densely populated regions during the recent COVID-19 pandemic, when CO_2_ emissions were drastically reduced by a combined halt of global transportation and industrial activities (van Heerwaarden et al. 2021). If the balance between atmospheric CO_2_ concentration and its solar dimming effect will be drastically disproportionated in the future, local heatwaves and enhanced water demand will be expected to severely affect agricultural productivity.

A realistic solution to this scenario could be offered, again, by biotechnological approaches. It is suggested that the content of photosynthetic pigments (chlorophylls) in plants could be lowered without affecting their growth performance (Genesio et al. 2021). However, the resulting pale green phenotype is expected to reflect a higher proportion of incident light compared with fully green varieties, determining a lower emission of heat from absorbed photons. This effect would reduce transpiration in plants (Sakowska et al. 2018) and yet would also limit air warming (Genesio et al. 2020) (Table 1). So far, the feasibility of this strategy has only been documented on small-scale field trials. However, it seems safe to predict that the widespread implementation of pale green crops would have a beneficial impact on agricultural output as well as play a role in the mitigation of global environmental changes.

**Table 1:** Summary table describing biotechnological approaches to enhance CO_2_ fixation, water- and light-use efficiency in plants

Aim	Targeted physiological process	Strategy	Host species	References
Enhancement of CO_2_ fixation	Carbon concentrating mechanism/leaf anatomy	Reprogramming of gene expression networks (ongoing)	Rice (*Oryza sativa*)	(Covshoff and Hibberd 2012; Von Caemmerer et al. 2012)
Enhancement of CO_2_ fixation	RuBisCo oxygenating activity	Introduction of cyanobacterial RuBisCo isoform	Tobacco (*Nicotiana tabacum*)	(Lin et al. 2014)
Enhancement of CO_2_ fixation	Photorespiration (glycolate metabolism)	Engineering of synthetic glycolate detoxifying pathways	Tobacco (*Nicotiana tabacum*)	(South et al. 2019)
Enhancement of light use	Non-photochemical quenching of excess light energy	Acceleration of NPQ relaxation kinetics through	Tobacco (*Nicotiana tabacum*)	(Kromdijk et al. 2016)
Enhancement of plant reflectance	Chlorophyll biosynthesis/assembly of photosynthetic light harvesting complexes	So far verified using existing spontaneous mutants	Soybean (*Glycine max*)	(Genesio et al. 2020; Sakowska et al. 2018)
